# Neddylation tunes peripheral blood mononuclear cells immune response in COVID-19 patients

**DOI:** 10.1038/s41420-022-01115-0

**Published:** 2022-07-12

**Authors:** Marina Serrano-Maciá, Sofia Lachiondo-Ortega, Paula Iruzubieta, Naroa Goikoetxea-Usandizaga, Alexandre Bosch, Leire Egia-Mendikute, Borja Jiménez-Lasheras, Mikel Azkargorta, Félix Elortza, Diana Martinez-Redondo, Begoña Castro, Juan J. Lozano, Ruben Nogueiras, Juan Irure-Ventura, Javier Crespo, Asís Palazón, María Carmen Fariñas, Teresa C. Delgado, Marcos López-Hoyos, Maria L. Martínez-Chantar

**Affiliations:** 1 Liver Disease Laboratory, CIC bioGUNE-BRTA (Basque Research & Technology Alliance), Centro de Investigación Biomédica en Red de Enfermedades Hepáticas y Digestivas (CIBERehd), Derio Bizkaia, Spain; 2 Gastroenterology and Hepatology Department, Marqués de Valdecilla University Hospital, Clinical and Translational Digestive Research Group, IDIVAL, Santander, Spain; 3 Cancer Immunology and Immunotherapy Lab, CIC bioGUNE, Basque Research and Technology Alliance (BRTA), Bizkaia, Spain; 4 Proteomics Platform, Center for Cooperative Research in Biosciences (CIC bioGUNE), Basque Research and Technology Alliance (BRTA), Carlos IIINetworked Proteomics Platform (ProteoRed-ISCIII), 48160 Derio Bizkaia, Spain; 5 Histocell S.L., Bizkaia Science and Technology Park, Derio Bizkaia, Spain; 6 Bioinformatics Platform, Centro de Investigación Biomédica en Red de Enfermedades Hepáticas y Digestivas (CIBEREHD), Barcelona, Catalonia Spain; 7 Department of Physiology, Research Centre of Molecular Medicine and Chronic Diseases, University of Santiago de Compostela-Instituto de Investigación Sanitaria, Santiago de Compostela, Spain; 8 Centro de Fisiopatología de la Obesidad y Nutrición, Centro de Investigación Biomédica en Red, Santiago de Compostela, Spain; 9 Galician Agency of Innovation (GAIN), Xunta de Galicia, Santiago de Compostela, Spain; 10 Servicio Inmunología, Hospital Universitario Marqués de Valdecilla-IDIVAL, Facultad de Medicina, Universidad de Cantabria, Barcelona, Cantabria Spain; 11 Ikerbasque, Basque Foundation for Science, Bizkaia, Spain; 12 Servicio Enfermedades Infecciosas, Hospital Universitario Marqués de Valdecilla-IDIVAL, Facultad de Medicina, Universidad de Cantabria, Barcelona, Cantabria Spain

**Keywords:** Immunology, Inflammatory diseases, Proteomics

## Abstract

The COVID-19 pandemic caused by SARS-CoV-2 has reached 5.5 million deaths worldwide, generating a huge impact globally. This highly contagious viral infection produces a severe acute respiratory syndrome that includes cough, mucus, fever and pneumonia. Likewise, many hospitalized patients develop severe pneumonia associated with acute respiratory distress syndrome (ARDS), along an exacerbated and uncontrolled systemic inflammation that in some cases induces a fatal cytokine storm. Although vaccines clearly have had a beneficial effect, there is still a high percentage of unprotected patients that develop the pathology, due to an ineffective immune response. Therefore, a thorough understanding of the modulatory mechanisms that regulate the response to SARS-CoV-2 is crucial to find effective therapeutic alternatives. Previous studies describe the relevance of Neddylation in the activation of the immune system and its implications in viral infection. In this context, the present study postulates Neddylation, a reversible ubiquitin-like post-translational modification of proteins that control their stability, localization and activity, as a key regulator in the immune response against SARS-CoV-2. For the first time, we describe an increase in global neddylation levels in COVID-19 in the serum of patients, which is particularly associated with the early response to infection. In addition, the results showed that overactivation of neddylation controls activation, proliferation, and response of peripheral blood mononuclear cells (PBMCs) isolated from COVID-19 patients. Inhibition of neddylation, and the subsequent avoidance of activated PBMCs, reduces cytokine production, mainly IL-6 and MCP-1 and induce proteome modulation, being a critical mechanism and a potential approach to immunomodulate COVID-19 patients.

## Introduction

The coronavirus disease 2019 pandemics (COVID-19) produced by SARS-CoV-2 has been the most important event worldwide in the last 50 years. COVID-19 has caused a terrible impact on society and economy, but mainly on health, with 313 million cases and 5.5 million deaths worldwide. This highly contagious viral infection may produce pneumonia and an acute respiratory distress syndrome (ARDS) associated with high mortality [1, 2]. However, COVID-19 is not just a respiratory disease, it is also a multi-organ condition [1].

To date, the severe immune reaction or cytokine storm against the SARS-CoV-2 infection is one of the major side effects associated with a poor outcome [2]. As it happens with other viral infections, the innate immune response is the first barrier in the fight against the virus, secreting inflammatory cytokines such as interleukin 1 (IL-1), interleukin 6 (IL-6) and type I interferon (IFNs), and subsequently activating the adaptive immune response [2]. During the SARS-CoV-2 infection, overactivation of the immune cells leads them to invade mainly the pulmonary interstitium, causing acute lung injury [2]. In these cases, the main treatment is based on the downregulation of the immune system activation using immunomodulators, but they are not without adverse effects [3, 4]. Huge efforts are currently ongoing to find an effective treatment, such as anti-spike neutralizing monoclonal antibodies, and direct acting antivirals or JAK and IL-6R inhibitors, involved in the surveillance, treatment and prevention of SARS-CoV-2 [5, 6]. Therefore, a deeper understanding of the mechanism underlying SARS-CoV-2 infection is required to find new strategies and to define the different stages involved in the immune response to treat accordingly.

In the last years, many researchers have described the relevant role of the post-translational modification (PTM) known as neddylation in the activation of the immune response and regulation of viral infections [7]. Neddylation is a reversible PTM characterized by the covalent conjugation of a small peptide NEDD8 (neuronal precursor cell-expressed developmentally downregulated protein 8) to lysine of target proteins in order to modulate their stability, localization, activity and function [8, 9]. The neddylation process is a highly conserved PTM that allows the cell to respond and adapt quickly and efficiently to various stimuli [10]. Under physiological conditions, neddylation levels are low, but overactivation of neddylation takes place as a response to cellular stress or DNA damage [11, 12], such as during a viral infection, inflammation, or cancer [7, 13–15], changing the proteome homeostasis and affecting the metabolism and functionality of the immune cells [7, 16]. In fact, a pharmacological inhibitor, MLN4924, was developed which selectively acts on NEDD8 activating enzyme 1 (NAE1) by forming covalent adducts and inhibiting its activity [15]. Interestingly, the NEDD8 pathway has been linked to oxidative and proteotoxic stress [8, 12]. Hence, given that modulation of the redox balance takes place during a viral infection in order to tune the immune response [17], neddylation is already considered a key regulator for viral infections [13, 18–20].

Herein, we have addressed for the first time to our knowledge the importance of neddylation in modulating the proteome associated to the immune system against SARS-CoV-2. Our results show high neddylation levels in the serum of COVID-19 patients compared to subjects without SARS-CoV-2 infection. The level of neddylation positively correlated with immunoglobulin M against spike, suggesting that neddylation might be involved in the first stage of immune defense. Likewise, an upregulation of the neddylated proteome was observed in Peripheral Blood Mononuclear Cells (PBMC) from COVID-19 patients versus those derived from healthy individuals. Interestingly, the pharmacological inhibition of neddylation in vitro, by using MLN4924 (pevonedistat) [15], reduced the activation, proliferation, and cytokine secretion of PBMC from COVID-19 patients. Moreover, the global proteomics fingerprint of PBMCs from COVID-19 patients showed a modulation of defense response, translesion DNA synthesis, and negative regulation of type I interferon production pathways, while the inhibition of neddylation resulted in changes at RNA catabolism, ER-associated processes, and viral genes, which might potentially explain the observed immunosuppressive phenotype.

Overall, we propose that neddylation may play a role adjusting the immune response against SARS-CoV-2 infection in COVID-19 patients, and that its dysregulation might be linked to a poor prognosis.

## Results

### COVID-19 patients show increased levels of serum NEDD8 proteins

**Fig. 1:**
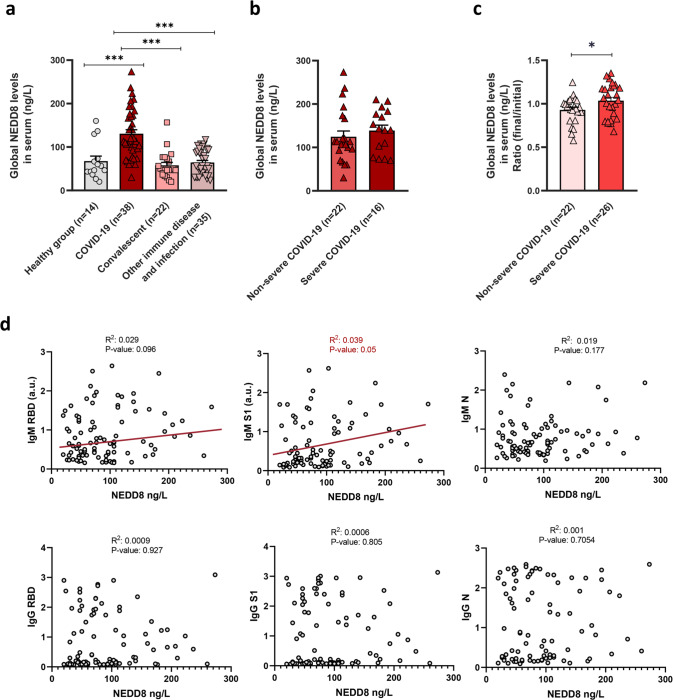
Serum global neddylation levels were increased in COVID-19 patients and positively correlated with IgM levels. **a** Serum global neddylation levels assessed by ELISA in a cohort of healthy subjects (*n* = 14), COVID-19 patients (*n* = 38), convalescents of COVID-19 disease (*n* = 22), patients with another non-treated immune disease such as systemic lupus erythematosus (SLE) and hepatitis C virus (HCV) (*n* = 37). **b** Serum global neddylation levels were evaluated between two groups of COVID-19 severity, non-severe COVID-19 (*n* = 22) and severe COVID-19 (*n* = 16). **c** Ratio between global neddylation levels from the final time of discharge and initial time of hospitalization in the second cohort of COVID-19 patients divided by the severity of the disease progression, moderate (*n* = 22) and severe (*n* = 26). **d** Correlation graphics between serum NEDD8 levels and IgM/IgM levels against SARS-CoV-2 Spike 1 (S1), receptor-binding domain (RBD) and nucleoprotein (N) evaluated from the first cohort of patients represented as *R*^2^ and *p*-value. Data are shown as average ± SEM and One-way ANOVA test was used to compare groups. ^∗^*p* < 0.05, ^∗∗^*p* < 0.01 and ^∗∗∗^*p* < 0.001 are shown.

The relevance of neddylation in COVID-19 has not been previously addressed. Thus, we evaluated if levels of serum NEDD8 proteins were differentially modulated in COVID-19 patients. Active COVID-19 patients showed a significant increase in serum NEDD8 levels in comparison with healthy individuals as well as to other patients with immune or chronic infection diseases, such as systemic lupus erythematosus or Hepatitis C virus (Fig. 1a and Supplementary Table 1). Importantly, in convalescent COVID-19 patients, the levels of NEDD8 remained lower, suggesting that neddylation was solely modulated in individuals with active SARS-CoV-2 infection. However, a deeper analysis showed no statistically significant differences in the serum NEDD8 levels of active patients either with moderate or severe disease (Fig. 1b).

Deciphering the dynamic changes of NEDD8 is important to understand its role in the immune response of COVID-19 patients. Analysis of ratio final vs initial serum NEDD8 levels showed an increased ratio in severe COVID-19 patients (Fig. 1c and Supplementary Table 2). These results suggest that the ability to regulate neddylation plays an important role in the disease progression.

In order to assess if the observed increase in the levels of serum NEDD8 levels were a direct consequence of a recent SARS-CoV-2 infection, we evaluated the presence of antibodies against SARS-CoV-2 in plasma from COVID-19 patients. Serum global NEDD8 levels showed a positive correlation with IgM against SARS-CoV-2 Spike (S1), while no differences were observed in IgM/IgG against virus antigens receptor-binding domain (RBD) and nucleoprotein (N) (Fig. 1d). As IgM provides the initial line of defence during viral infections, this result may suggest that neddylation would be involved in the first step of adaptive immune response in SARS-CoV-2 infection.

Thus, augmented serum NEDD8 levels were associated with an active immune response against SARS-CoV-2 infection in COVID-19 patients.

### Modulation of neddylation alters the immune response in primary peripheral blood mononuclear cells (PBMCs)

**Fig. 2:**
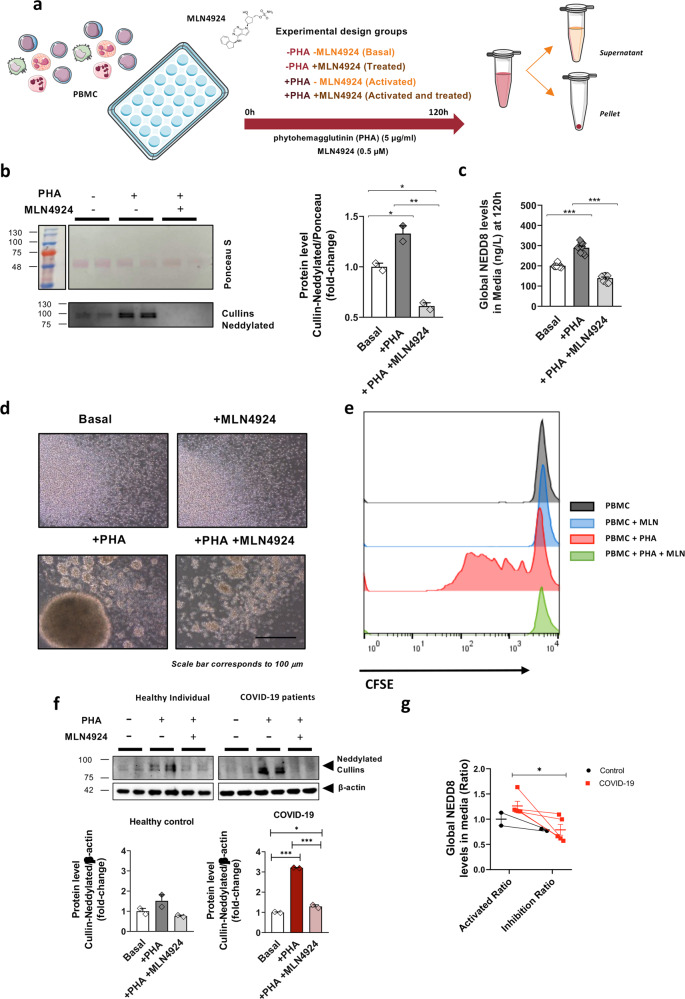
Neddylation levels tune the immune response in primary peripheral blood mononuclear cells (PBMCs) from healthy donors and COVID-19 patients. **a** PBMCs in vitro experimental design using mitogen phytohemagglutinin (PHA) (5 µg/ml) as an immune system activation, and MLN4924 (0.5 µM) as a neddylation inhibition during 120 h. 4 groups of study were designed using the combination of the present treatments. **b** Protein levels of NEDD8-Cullins conjugated, ponceau and quantification form PBMCs from healthy donor, activated with PHA and activated with PHA and treated with MLN4924. **c** Global Neddylation levels in culture media form PBMCs from healthy donor, activated with PHA and activated with PHA and treated with MLN4924. **d** PBMCs pictures 120 h after treatments from 4 treatment groups. **e** Assessment of proliferation of PBMCs from 4 groups of treatments with Carboxyfluorescein succinimidyl ester (CFSE) by flow-cytometry. **f** Protein levels of NEDD8-Cullins conjugated, ponceau staining and quantification in PBMCs from healthy donors and COVID-19 patients. **g** Global Neddylation levels in culture media in PBMCs from healthy donors or COVID-19 patients calculated by activation ratio as a basal state versus activation with PHA, and inhibition ratio calculated by activation with PHA versus Activated and treated with MLN4924. Scale bar corresponds to 100 μm. Data are shown as average ± SEM and One-way ANOVA test and *T*-test respectively, was used to compare groups. ^∗^*p* < 0.05, ^∗∗^*p* < 0.01 and ^∗∗∗^*p* < 0.001 are shown. Symbols description: − without and + with.

To better understand the relationship between NEDD8 levels and the immune response, PBMCs from healthy donors were activated with a mitogen phytohemagglutinin (PHA) treatment. PBMC activation resulted in higher levels of NEDD8, both in the cell lysates and in the secretome, evaluated in the culture medium. Moreover, the levels of neddylated cullins, the most well-described substrate of NEDD8, were also induced under these conditions (Fig. 2a–c).

MLN4924 is a reported as a specific inhibitor of the first enzyme of the neddylation pathway, NEDD8 activating enzyme (NAE-1) [15]. In order to evaluate the impact that neddylation inhibition could have in the immune response, PBMCs from healthy individuals were exposed to PHA in the presence or absence of MLN4924. Treatment with MLN4924 efficiently reduced the levels of neddylated cullins and significantly decreased NEDD8 levels in cell extracts and media (Fig. 2b, c). Under these circumstances, active proliferative PMBCs acquired phenotypic characteristics that were completely reversed in the presence of MLN4924 (Fig. 2d). Evaluation of the cell proliferation based on CFSE dilution by flow cytometry analysis also showed that the absence of neddylation reduced the proliferative capacity of activated PMBCs (Fig. 2e). Under these conditions, MLN4924 did not induce an apoptotic response, rather maintaining the PBMCs in a quiescent state (Supplementary Fig. 1a).

Importantly, a comparative study was performed in PBMCs from COVID-19 patients versus healthy individuals (Supplementary Table 3). As expected from our previous data, the level of neddylated cullins was higher in stimulated COVID-19 PBMCs than in stimulated healthy control PBMCs. Indeed, inhibition of NAE-1 by MLN4924 resulted in a more significant response in intracellular NEDD8 levels and neddylated secretome analyzed by both western blot and ELISA in COVID-19 patients than in samples from healthy individuals (Fig. 2f, g).

Overall, these data confirm that neddylation may be involved in modulating the immune response, which is increased in COVID-19 patients, and that its inhibition reduces the proliferation and activation of PBMCs.

### Cytokine profile from PBMC-COVID-19 patients under neddylation inhibition

**Fig. 3:**
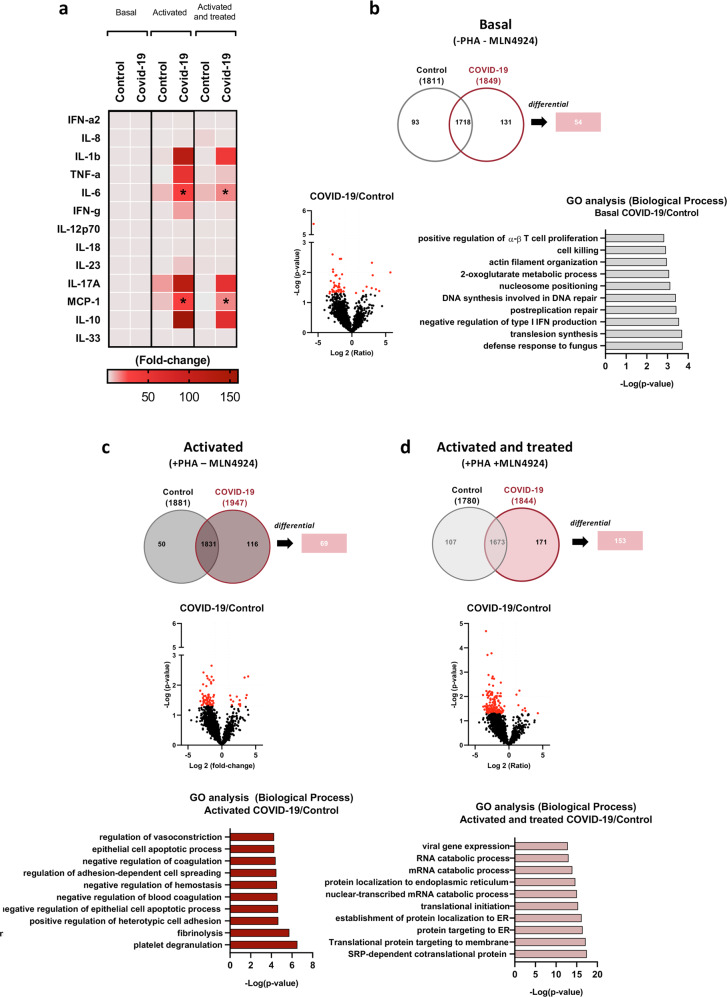
Neddylation regulates cytokines secretion, and change proteome in PBMCs from healthy donors and COVID-19 patients. **a** Cytokine quantification in culture media from PBMCs from healthy donor and COVID-19 patients represented as fold-change in Heat Map graphics. **b** Proteomic analysis comparation between basal PBMCs represented as a Van der Venn diagram, volcano plot and Gene Ontology overrepresented biological process from significative differential proteins in COVID-19 patients over healthy donor. **c** Proteomic analysis comparation between Activated with mitogen phytohemagglutinin (PHA) PBMCs represented as a Van der Venn diagram, volcano plot and Gene Ontology overrepresented biological process from significative differential proteins in COVID-19 patients over healthy donor. **d** Proteomic analysis comparation between Activated with PHA and treated with MLN4924 PBMCs represented as a Van der Venn diagram, volcano plot and Gene Ontology overrepresented biological process from significative differential proteins in COVID-19 patients over healthy donor. One-way ANOVA test was used to compare groups. ^∗^*p* < 0.05, ^∗∗^*p* < 0.01 and ^∗∗∗^*p* < 0.001 are shown. Symbols description: −/− PBMC group without PHA and MLN4924; +/− PBMCs group with PHA and without MLN4924; +/+ PBMCs group with PHA and MLN4924.

The cytokine response following SARS-CoV-2 infection has become a key issue in the COVID-19 patients. In order to understand how neddylation modulation may influence the cytokine secretome of the PBMCs derived from COVID-19 patients and healthy individuals after PHA stimuli and in the presence or absence of MLN4914 treatment, by employing flow cytometry approaches. Cytokine heatmap revealed that COVID-19 patients presented an overstimulation of these molecules specially IL-6 and monocyte chemoattractant protein-1 (MCP-1) (Fig. 3a) respect to basal state. Blocking neddylation with MLN4924 resulted in a significant reduction of these two cytokines in the PBMCs derived from COVID-19 patients, while a non-significant response was identified in healthy individuals (Fig. 3a) respect activated state. Therefore, neddylation has a significant impact in the cytokine production involved in immune system activation in COVID-19 patients.

Therefore, inhibition of neddylation might play a role in modulation Il-6 and MCP-1, which have a significant impact on the immune response of COVID-19 patients [21–23].

### Neddylation modulates the proteomic profile of COVID-19 PBMCs patients

To interrogate the homeostasis of the proteomic profile from COVID-19 and control PBMCs associated to neddylation inhibition, high-throughput proteomics was performed in PBMCs isolated from healthy controls or COVID-19 patients. The Venn diagram representation showed significantly different 54 proteins between basal-state, healthy versus COVID-19 PBMCs (Fig. 3b). Besides, the Volcano plot resulting from the proteomics profile, exhibited that most of them were regulated in the PBMCs from COVID-19 patients (Fig. 3b). The Gene Ontology Enrichment Analysis was used to identify the major pathways involved in the response that SARS-CoV-2 produced in the cells. The results showed that processes such as the defense response, translesion synthesis and negative regulation of type I interferon production were overrepresented in the PBMCs coming from COVID-19 patients, (Fig. 3b), in line with a possible basal priming. A heat map indicating the top 50 proteins regulated in cells derived from these patients is shown in Supplementary Fig. 2a. A file containing all the identified proteins is provided in Supplementary File 1.

To further understand the different mechanisms underlying the activation of the PBMCs derived from COVID-19 patients and healthy controls, a proteomic profile was performed after the treatment with PHA for 48 h. The Venn diagram identified 69 differential proteins (Fig. 3c), and the Gene ontology analysis described those pathways like platelet degranulation, fibrinolysis and cell adhesion were overrepresented in PHA-activated PBMCs from COVID-19 patients (Fig. 3c). The Heat map of the top 50 proteins that are differentially modulated between both groups is shown in Supplementary Fig. 2b.

**Fig. 4:**
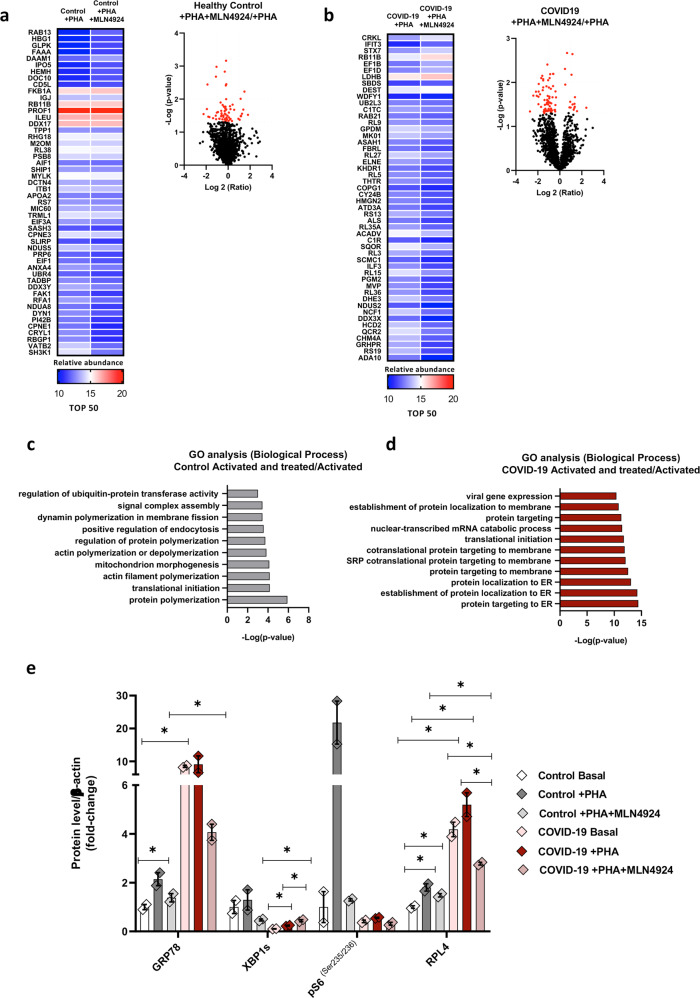
Neddylation inhibition modulate proteome in PBMCs from healthy donor and COVID-19 patients. **a** Proteomic analysis comparation from PBMCs activated with mitogen phytohemagglutinin (PHA), and activated and treated with MLN4924 from healthy donors represented as a top 50 heat map and volcano plot**. b** Proteomic analysis comparison from PBMCs activated with PHA, and activated and treated with MLN4924 from COVID-19 patients represented as a top 50 heat map and volcano plot. **c** Gene Ontology analysis of overrepresented biological process from significative differential proteins in PBMCs activated and treated with MLN4924 from healthy donor. **d** Gene Ontology analysis of overrepresented biological process from significative differential proteins in PBMCs activated and treated with MLN4924 from COVID-19 patients. **e** Protein quantification of Glucose-Regulated Protein 78 kDa (GRP78), X-Box Binding Protein 1 spliced (XBP1s), phosphorylation of S6 ribosomal protein (Ser235/236), Ribosomal protein L4 (RPL4) and β-actin form PBMCs from healthy donor and COVID-19 patients in basal state, activated with PHA and activated with PHA and treated with MLN4924. Data are shown as average ± SEM and *T*-test was used to compare groups. ^∗^*p* < 0.05, ^∗∗^*p* < 0.01 and ^∗∗∗^*p* < 0.001 are shown.

Once we identified the proteomic profile associated with the basal and active states of PBMCs from COVID-19 patients and healthy individuals, we analysed the effects of neddylation blockade. A total of 153 significantly regulated proteins were identified in PMBCs of COVID-19 patients compared to cells derived from healthy individuals when neddylation was inhibited (Fig. 3d). The resulting proteomic profile after GO analysis showed pathways implicated in viral regulation, RNA catabolic processes that implies breakdown of mRNA, translation initiation and stimulation of ER stress-related. Importantly, the comparison between PBMCs from COVID-19 patients activated and in presence or absence of MLN4924 render a total of 84 proteins that appeared modulated. While no significant differences were observed in PBMCs treated with the inhibitor of neddylation in PHA-activated cells from healthy individuals (Fig. 4a, b).

As expected, the pathways regulated by MLN4924 in the PBMCs derived from COVID-19 patients coincided with those previously described, including RNA catabolism, ER-associated processes and expression of viral genes. However, in the case of the PBMCs derived from healthy individuals, the inhibition of neddylation coincided with a regulation of the processes involved in ubiquitination, due to the effect of MLN4924 on the inhibition of Cullin-RING E3 ubiquitin Ligase activity (CRLs) and therefore onto proteasomal degradation [24]. Accordingly, regulation of protein polymerization associated to modulation of CRLs was one of GO pathways identified in these cells (Fig. 4c, d).

To further confirm the signalling pathways modulated under these experimental conditions, PBMC from healthy subjects and COVID -19 patients were analysed by western blot analysis (Fig. 4e and Supplementary Fig. 2d). In association with ER stress, levels of GRP78, the major chaperone protein critical for the stability of these proteins were significantly increased at basal levels in PBMCs from COVID-19 patients compared with cells from healthy subjects (Fig. 4e and Supplementary Fig. 2d). However, XBP1, spliced protein related to IRE1 activation [25], was significantly reduced at basal levels in PBMCs from COVID-19 patients compared with cells from healthy subjects (Fig. 4e). Importantly, PHA elicited a stress response that was significantly improved by MLN4924 treatment, leading to a marked reduction in GRP78 (Fig. 4e and Supplementary Fig. 2d). Moreover, S6 activity, as measured by S6 phosphorylation at residues Ser235 and 236, appeared to be significantly inhibited in PBMC from SARS-CoV-2 patients, as previously described [26]. Importantly, inhibition of neddylation abrogated S6 activation, which was shown after PHA treatment in PBMC from healthy individuals (Fig. 4e and Supplementary Fig. 2d).

Finally, to evaluate the modulation of translation in PBMCs from COVID -19 and healthy subjects, the concentration of RPL4 was measured in these cells. Importantly, RPL4 was induced in SARS CoV-2 infected PBMCs compared with control cells under resting conditions. PHA induced the expression of RPL4, whereas MLN4924 blocked this response (Fig. 4e and Supplementary Fig. 2d). These results are consistent with the modulation of ER stress detected under these conditions.

These data indicate a significant induction of the ER stress response in PBMC from COVID-19 patients, even at basal levels and in association with a higher level of neddylation, making these cells more susceptible to NAE-1 inhibition.

## Discussion

Although more than 8 billion doses of SARS-CoV-2 vaccines have been administered around the world, no vaccine is completely effective at preventing symptomatic cases of COVID-19 [27, 28]. Therefore, a wide comprehension of the mechanism involved in SARS-CoV-2 infection and immune system alteration is critical to find effective therapeutic alternatives.

As aforementioned, the neddylation process a highly conserved post-translational modification is thought to control viral amplification and replication, mainly by controlling the production of type I interferon [13]. Neddylation not only regulates viral infectivity, but also acts on the innate immune response and regulating T lymphocyte activity [7]. Thus, neddylation plays a fundamental role in the inflammatory response. For example, neddylation inhibition in LPS-triggered proinflammatory processes, blocks the production of proinflammatory cytokines that depend mainly on the NF-κB pathway, including IL-6 and TNF among others [29]. Based on these premises, we described the importance of neddylation, a reversible PTM, for immune activation in COVID-19 patients and the potential efficacy of MLN4924, the pharmacological inhibitor of neddylation. We have recently described that neddylated proteins appear in the serum of patients with liver pathology, which is associated with disease progression [30]. High levels of proteins in the blood could be a sign of chronic infection or inflammation [31]. In this study, we show for the first time that patients with active COVID-19 have significantly increased serum NEDD8 protein levels, which are, indeed, positively correlated with serum IgM levels. Importantly, NEDD8 levels were not modulated in chronic viral hepatitis C or SLE, suggesting a specific acute response in COVID-19 patients. Serum NEDD8 levels also seem to be linked to disease progression. At the time of hospitalization both groups presented similar serum NEDD8 levels. However, the comparison of final vs. initial serum NEDD8 levels showed an increased ratio in severe patients, hinting the ability of non-severe patients to downregulate the neddylation conjugates, and, thereby, halt disease progression. Related to the effect of neddylation in the immune response, we found that compared to healthy individuals, PBMCs coming from COVID-19 patients had a significantly higher content of neddylated proteins, both at intracellular and secretome levels. These results suggest a significant relationship between neddylation and active SARS-CoV-2 infection in COVID-19 patients and its possible outcome. Moreover, the accumulation of neddylated proteins in PBMCs could play an important function in the activity of these cells, and their overrepresentation in the culture media and serum could serve as an indirect way to modulate the crosstalk between immune cells.

It is important to highlight that PBMCs from COVID-19 patients not only showed higher levels of neddylated proteins but also, the levels of this PTM upon PHA activation further increased compared to activated healthy PBMCs. The susceptibility for increased NEDD8 levels could be due to the prior exposure with the infected cells. Neddylation thus appears to be a response to the immunological stress that SARS-COV2 would exert on these cells. In this context, it is important to note that blocking neddylation with MLN4924 induced cycle arrest in activated PBMCs. These results agreed with previous data regarding the role of neddylation in T cell function; neddylation was found to modulate the proliferative response and cytokine production through the inhibition of Ubc12 [32], it also controls the different T cell subtypes as well as differentiation into Tregs [33] and finally, it can regulate the T cell response by modulating PD-L1 levels via a cullin-dependent mechanism [34].

Production of proinflammatory cytokines by immune cells is required to drive the immune response during SARS-CoV-2 infection. However, a persistent inflammatory state can lead to a severe systemic damage. When cytokine levels were evaluated in the culture media of PBMCs derived from healthy individuals and COVID-19 patients, a higher tendency to secrete cytokines such as IL-1b, TNF, IL-6, INFg, IL-17, MCP-1, and IL-10 was observed in cells exposed to SARS-COV2 infection. Inhibition of neddylation by MLN4924 was able to reduce some cytokines in PBMCs from COVID-19 patients, while no effect was observed in controls. Thus, only cells previously exposed to SARS-COV2, with a high intracellular and extracellular NEDD8 content, responded to the inhibition of neddylation. Moreover, a particular effect has been noted with IL-6 and MCP-1. IL-6, produced by various immune cell types and a well-known mediator of the proinflammatory response, and MCP-1, involved in the infiltration and migration of monocytes, have previously been associated with COVID-19 patients and the disease severity [35]. Serum MCP-1 levels have also been linked to a higher risk of thrombosis and platelet regulation in patients infected with SARS-CoV-2 [36]. Therefore, the results obtained suggest that the neddylation pathway may play a role in immune activation in COVID-19.

To understand proteome homeostasis modulated by neddylation in PBMCs from COVID-19 patients and healthy individuals, high-throughput proteomic analysis was performed in these cells comparing fingerprints in the basal state, under activated stimuli, and in response to neddylation inhibition. Gene Ontology Proteomic analysis revealed an exacerbated state of PBMCs from COVID-19 patients in which pathways involved in the defence response, translation regulation, and negative regulation of interferon in basal state were overrepresented. These results reflect the intrinsic proteome modulation after the SARS-COV2 infection and agree with the activated state of PBMCs from COVID-19 patients. However, after the activation of the PBMCs, COVID-19 patients proteomics showed an overrepresentation of pathways related with platelets, fibrinolysis, and cell adhesion. According to previous publications, thrombo-inflammation processes related to platelets levels were a risk factor for COVID-19 pathogenesis [37, 38]. Hence, the regulation of these pathways in COVID-19 patients are intrinsic of SARS-CoV-2 infection and it is not associated with PBMCs activation.

Likewise, the pharmacological blockage of neddylation, using MLN4924 in activated PBMCs, induced a proteome reorganization. PBMCs from COVID-19 patients showed an overrepresentation of pathways related to the endoplasmic reticulum and RNA catabolic processes. Indeed, viral infections induce an excessive production of reactive oxygen species (ROS), which cause endoplasmic reticulum stress [17]. The ER is strongly involved in the proper folding and trafficking of proteins, via unfolded protein response (UPR) system, as well as in the regulation of cytokine production, processing, and secretion, especially in the immune system [39]. Besides, the ER stress is known to induce inflammatory responses through the UPR pathway, and immune cells are highly dependent on the UPR signalling pathways to develop their specific functions. In fact, new therapies based on targeting the UPR are investigated as a treatment for immune disorders [40]. Therefore, a correct function of this organelle is essential for the activation, functionality, and maturation of diverse immune cells. Based on our results, we suggest that neddylation inhibition can repress protein translation and, consequently, decrease the protein synthesis related with ER stress [39–42]. Therefore, we can explain how the activation with PHA induced the endoplasmic reticulum stress that MLN4924 is able to alleviate, by reducing protein translation, along with the production and processing of cytokines.

Finally, neddylation inhibition in the PBMCs from the healthy group presented a weak proteome modulation when compared to active PBMCs, indicating that MLN4924 does not induce a strong change in these conditions. Following the blockade of neddylation, regulation of ubiquitination was an overrepresented pathway in the GO analysis, due to the relevance of neddylation in the Cullin-Ring ligase E3 activity. However, the pharmacological treatment of activated COVID-19 patients’ PBMCs with MLN4924, showed a strong proteome modulation with overrepresented pathways such us mRNA catabolic processes, ER-associated processes, and expression of viral genes. Importantly, the common pathway overrepresented in both groups was the initiation of translation. The analysis showed that MLN4924 is targeting RNA translation, via modulating the destabilization of ribosomal proteins as Xirodimas et al., 2008 already reported [43]. Therefore, immune cells are incapable to synthetize new proteins, and subsequently, unable to be activated and proliferate, what may explain the immunosuppressive effect of MLN4924.

In this sense, an induction of markers related to ER stress, such as GRP78 and XBP1, was identified in PBMCs from COVID-19 patients compared to healthy subjects at basal levels. Importantly, inhibition of neddylation significantly attenuate this stress response. Accordingly, translation activity was also measured by the expression of RPL4 marker that was significantly induced in PBMC from COVID-19 patients accordingly with the ER activity and modulated under MLN4924 induction. Finally, S6 phosphorylation was significantly inhibited in PBMCs from COVID-19 patients, consistent with the results previously described in the literature with the metabolic rewiring in mTOR metabolism [26].

Altogether, our results prove that neddylation is a key pathway involved in the immune response against SARS-COV2 infection. Indeed, we show that serum NEDD8 levels are significantly augmented and specifically associated with COVID-19 patients during the early response to the infection. Moreover, activated PBMCs derived from SARS-CoV-2 infected individuals show increased neddylation, whereas its inhibition and concomitant reduced PBMCs activation, assessed both by cytokine production and proteomic analysis, may be a critical mechanism to immunomodulate COVID-19 patients and, probably, avoid progression to a fatal outcome from a dysregulated systemic inflammatory response.

## Materials and methods

### Human samples

The human serum samples that have been used in this work were obtained after an informed consent of the subjects and in accordance with the Ethical Code of the World Medical Association and Helsinki Declaration and upon approval of the Hospital Marques de Valdecilla (Santander, Spain) ethics Committee (CEIm, internal code 2020.167, 14 May 2020).

Five well-characterized cohorts of subjects were used in the present study. The first cohort included healthy subjects (*n* = 14, 35.7% women and 64.3% men, mean age 61 ± 11) without any infectious or inflammatory pathology. The second cohort included patients with systemic lupus erythematosus (SLE) or chronic hepatitis C virus infection (*n* = 35, 49% SLE and 52% hepatitis C, 60% women and 40% men, mean age 50 ± 15). Both cohorts were used as controls and recruited by the Gastroenterology and Hepatology Department and Immunology Department of Marqués de Valdecilla University Hospital. The third cohort included convalescent patients one month after the clinical onset of the COVID-19 (*n* = 22, 50% women and 50% men, mean age 50 ± 17). The fourth cohort was comprised by patients with laboratory-confirmed COVID-19 who were hospitalized at Marqués de Valdecilla University Hospital between March and May 2020 (*n* = 38, 47.4% women and 52.6% men, mean age 58 ± 14; complete information in Supplementary Table 1). Serum was obtained from all COVID-19 patients on the day of hospitalization. Furthermore, the final cohort was comprised by 48 COVID-19 patients serum samples drawn since the hospitalization time until discharge (*n* = 22, 50% women and 50% men, mean age 50 ± 17; complete information in Supplementary Table 2). COVID-19 severity was evaluated during hospitalization and divided into 4 subtypes: mild, moderate, severe and critical illness according to management guidelines in China. We defined mild and moderate COVID-19 subtypes as ‘non-severe COVID-19, and severe and critically ill subtypes as ‘severe COVID-19 [29]. All patients received standard medical treatment according to the COVID-19 management guidance.

Finally, PBMCs were isolated from blood samples of 5 COVID-19 patients at the moment of hospitalization. A summary of the clinical data of COVID-19 patients is shown in Supplementary Table 3.

### NEDD8 ELISA assay

Global neddylation levels in human serum (15 µl) and culture media (50 µl) of human PBMCs were analyzed using an ELISA kit that specifically recognizes the human NEDD8 sequence (MBS1606634, MyBiosource) according to the instructions.

### Quantification of ELISA (immunoglobulin G and M) against the immunogenic viral antigens

The protocol to quantify the serum levels of IgG and IgM against the immunogenic viral antigens Receptor-Binding Domain (RBD), Spike (S) and Nucleoprotein (N) was performed as described in Egia-Mendikute et al., 2020 [44].

### Peripheral blood mononuclear cells (PBMCs) purification

Peripheral Blood Mononuclear Cells from peripheral blood were purified following the protocol described in Panda S et al., 2012 [45].

### PBMCs in vitro treatments

Purified PBMCs were defrosted, and after evaluating the viability, they were counted and plated: 150.000 cell per well in muti-well plates with DMEM Glutamax, 10% of FBS and 5% of antibiotic-antimycotic mix. Finally, Phytohemagglutinin (L4144 Sigma), to induce the activation of the immune cells (PHA; 5 µg/mL), and MLN4924, to inhibit neddylation (Pevonedistat; 0.5 µM) (MeDChemExpress), were added to the corresponding groups and plates were incubated at 37 °C with 5% CO_2_. Four groups were used to evaluate the effects of MLN4924 in PBMC; control group (PHA- MLN-), control group treated with MLN4924 (PHA-MLN+), activated group (PHA + MLN-), activated and treated group (PHA+MLN+). Pictures were taken and the supernatant and pellet were collected at 0, 24, 48, 72, 96 and 120 h.

### Proliferation assay with CFSE by flow cytometry

Purified PBMCs were stained with 0.8 µM of carboxifluorescein diacetate succinimidyl ester (CFSE; (CellTrace, C34554 ThermoFisher) has been stablished as a very useful tool that provides information about cell proliferation [46].

After 96 and 120 h, cells were collected and subsequently stained with propidium iodide (PI; Life Technologies, P3566) (50 µg/mL) in order to identify the living cells by flow cytometry. Data acquisition and analyses were performed in a flow cytometer (Guava EasyCyte, Merk Millipore) using GuavaSoft 3.2 software and at least 3000 events were collected for each sample. Results obtained were finally analysed in FlowJo (BD).

### Protein isolation and western blotting

The protocol for protein isolation and western blotting was described in Serrano-Maciá et al., 2021 [30]. Protein gels were transferred into nitrocellulose blotting membranes. These were blocked with 5% non-fat milk in TBS PH 8 containing 0.1% Tween-20 (Sigma Aldrich) (TBST-0.1%) for 60 min at RT, washed 3 times with TBST and incubated overnight at 4 °C with commercial antibodies for NEDD8 (ab81264), Mouse Anti-BiP/GRP78 (610978, BD), Anti-XBP1 antibody (ab37152, Abcam), Phospho-S6 Ribosomal Protein (Ser235/236) (91B2) (#4857, Cell Signalling), Ribosomal Protein L4 (RQ-7) (sc-100838, Santa Cruz) and β-actin (a5441). They were then incubated with the secondary antibodies conjugated with horseradishperoxidase (HRP) for 1–2 h RT (Anti-mouse IgG c7076s and anti-rabbit-IgG-HRP-linked c7074, Cell Signalling). Immunoreactive proteins were detected by using ECL substrate on an iBright system. Band densitometry was performed using ImageJ.

### Cytokines quantification assay

Total quantification of cytokines from PBMC supernatant was performed using the LEGENDplex inflammation panel 1 ELISA (BioLegend, Cat. 740809) and flow cytometry (BD Biosciences). Data was acquired on FACSymphony flow cytometer (BD Biosciences) and results were analyzed according to the manual and the program provided by the manufacturer.

### Proteomics

#### Protein extraction and in solution digestion

PBMCs coming from 5 COVID-19 patients and 2 healthy control donors were analyzed in this work. Healthy control cells from each donor were analyzed in independent duplicates for a better readout of the control group. Protein was extracted following previous data [30]. Briefly, protein was extracted using 7 M urea, 2 M thiourea, 4% CHAPS. Samples were incubated for 30 min at RT under agitation and digested following the SP3 protocol described by Hughes et al. 2014 [47] with minor modifications. Trypsin was added to a trypsin:protein ratio of 1:10, and the mixture was incubated 2 h at 37 °C. After recovery, peptides were dried out in a RVC2 25 speedvac concentrator (Christ), and resuspended in 0.1% FA. Peptides were desalted and resuspended in 0.1% FA using C18 stage tips (Millipore) prior to acquisition.

#### Mass spectrometry analysis

Samples were analyzed in a hybrid trapped ion mobility spectrometry—quadrupole time of flight mass spectrometer (timsTOF Pro with PASEF, Bruker Daltonics) coupled online to a EvoSep ONE liquid chromatograph (EvoSep). Sample (200 ng) was directly loaded in a EVOSEP ONE chromatograph and acquired using the 30 spd protocol.

Protein identification and quantification was carried out using MaxQuant software [48] using default settings except for an LFQ min. ratio count of 1. Only proteins identified with at least two peptides at FDR < 1% were considered for further analysis. Data (LFQ intensities) was loaded onto Perseus platform [49] and further processed (log2 transformation, imputation). A *t*-test was applied and proteins with a *p* < 0.05 were considered for discussion.

#### Networks, functional and pathways mapping

Proteins with a significant *p*-value (*p* < 0.5) were submitted to a GO Enrichment Analysis using enrich GO function from cluster Profiler R package [50].

#### Statistical analysis

Prism 8 (GraphPad Software, version 8.4.0) was used to perform statistical analysis. One-way analysis of variance (ANOVA) followed by Tukey (comparing all pairs of columns) was used in case of three or more groups, while Student’s *t*-test was used in case 2 groups. Grubbs’ test was performed to determine the significant outliers. A *p* < 0.05 was consider statistically significance. Statistical parameters are reported in the figure legends.

## Supplementary information

Supplemental material

Original Data File

## Data Availability

Row data from proteomic identification and analysis is available with the accession number PXD033673 at PRIDE and ProteomeXchange database (http://proteomecentral.proteomexchange.org/cgi/GetDataset).
