# Supplementary material for: Neddylation tunes peripheral blood mononuclear cells immune response in COVID-19 patients
Source: Cell Death Discov. 2022 Jul 12;8:316. doi: 10.1038/s41420-022-01115-0 (PMC9277603; doi:10.1038/s41420-022-01115-0)
Supplement: Supplementary file 1 — Supplemental material [file 41420_2022_1115_MOESM1_ESM.docx]

**Supplemental material**

**
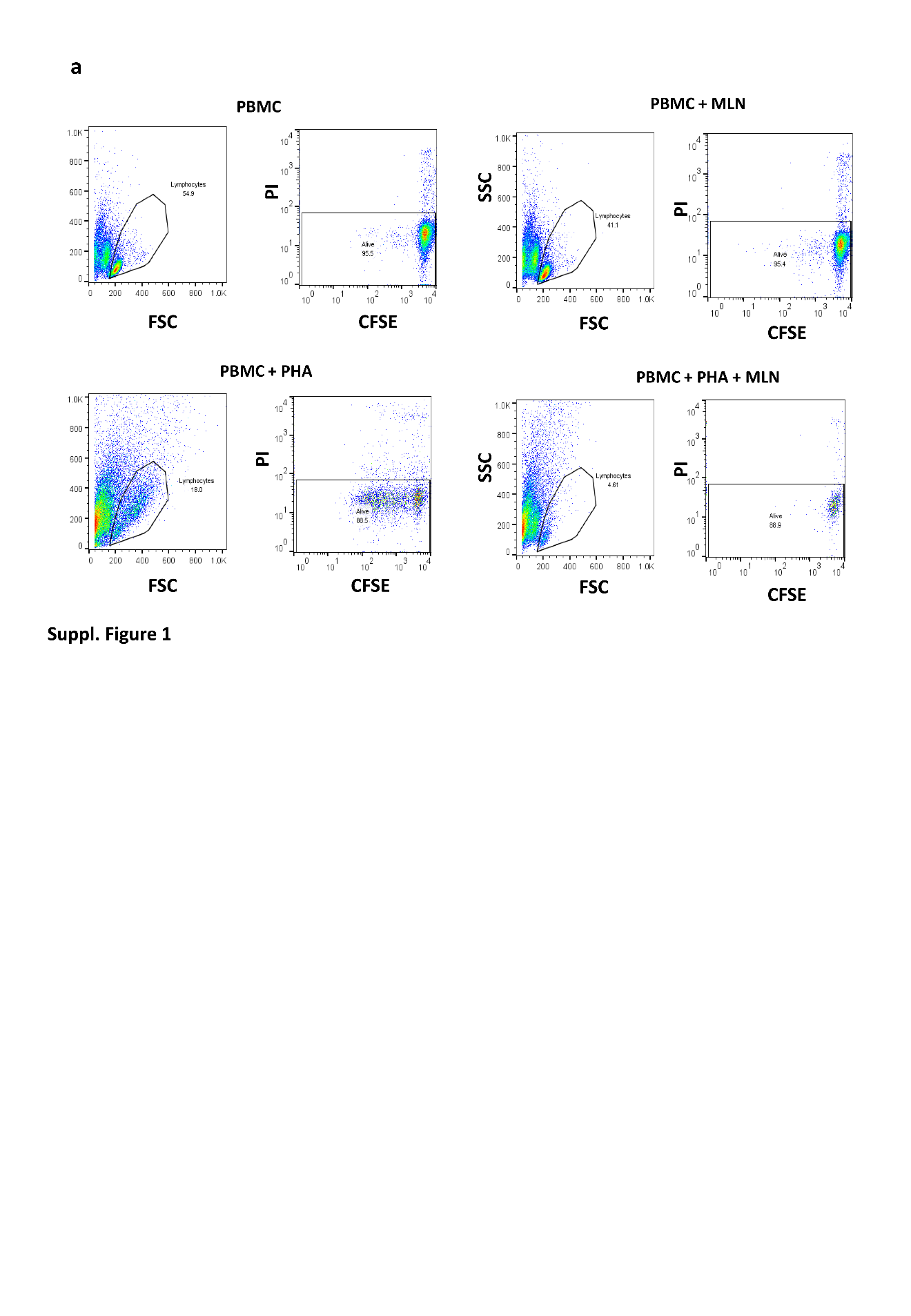
**

**Supplemental Figure 1: Proliferation are affected after activation and treatment with the pharmacological inhibition of neddylation in PBMC from healthy donor. a.** Forward scattering and death and live percentage form those 4 groups of PBMC analysed.


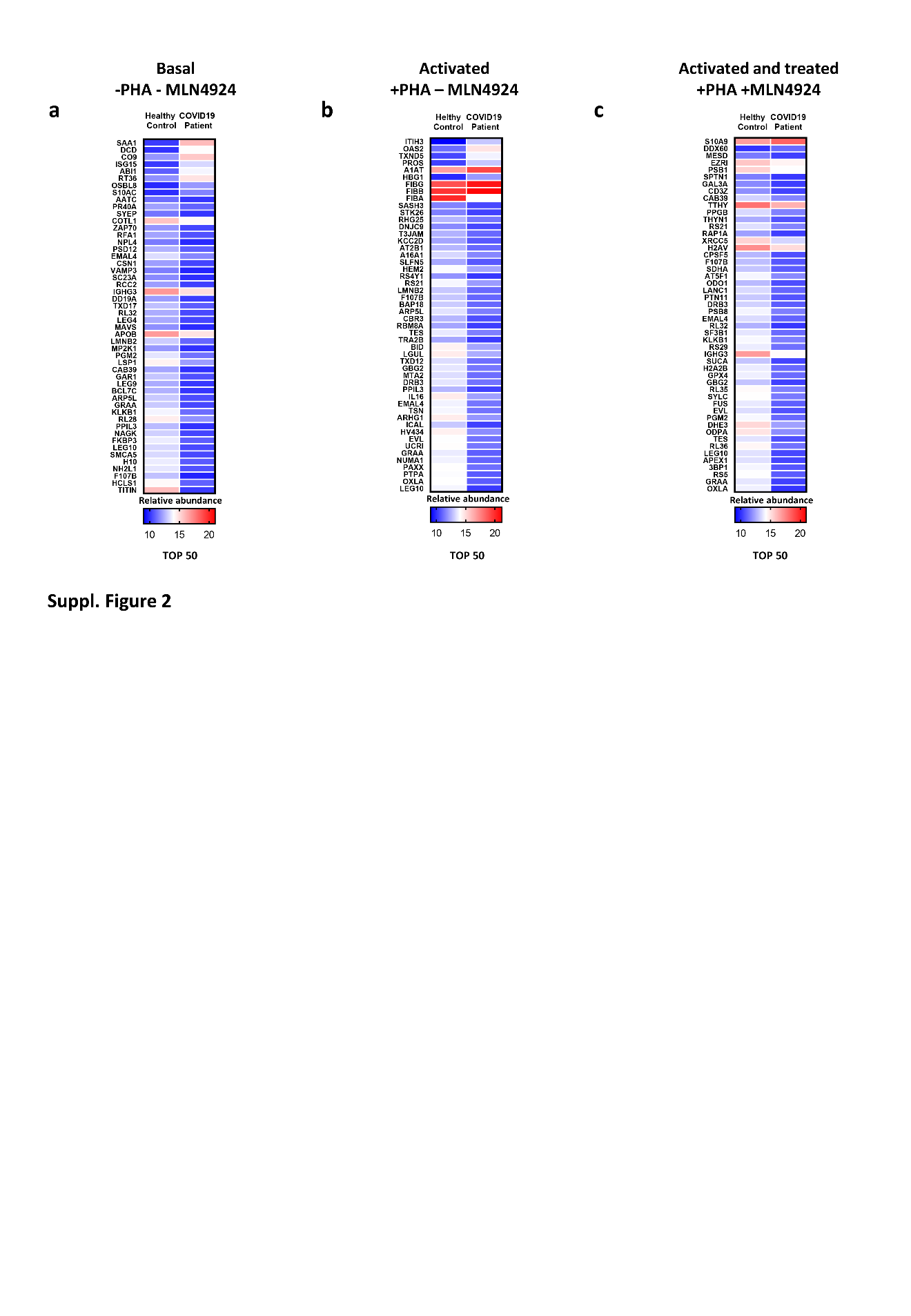


**Supplemental Figure 2: Proteomic characterization by Liquid Chromatography-Mass Spectrometry (LC-MS) in PBMC from healthy donor and COVID-19 patients. a.** Heatmaps showing the top 50 significantly different most differentially expressed proteins in COVID-19 patients over healthy donor in basal state. **b.** Heatmaps showing the top 50 significantly different most differentially expressed proteins in COVID-19 patients over healthy donor activated. **c.** Heatmaps showing the top 50 significantly different most differentially expressed proteins in COVID-19 patients over healthy donor activated and treated with MLN4924.

**Supplemental Table 1****: Summary of clinical data of COVID-19 patients from serum cohort 4.**

|  | COVID-19 Serum samples | |
| --- | --- | --- |
| n | 38 | |
| Age (years) | 58 ± 14 | |
| Gender (F/M) | 19/19 | |
| PCR + | 38 | |
| Mild (n) | 5 | |
| Moderate (n) | 17 | |
| Severe (n) | 6 | |
| Very severe (n) | 10 | |
| Obesity (n) | 15 | |
| Cardiopathy (n) | 4 | |
| Chronic Pulmonary disease (n) | 5 | |
| Chronic Renal disease (n) | 2 | |
| Immunosuppression (n) | 3 | |
| Cancer in last 5 years (n) | 1 | |
| Pneumonia (n) | 38 | |
| Hospitalization (n) | 32 | |
| ICU income (n) | 38 | |
| Respiratory distress | 10 | |
| VMNI | 13 | |
| IOT | 3 | |
| Exitus | 2 | |
| Vaccinated (n) | 0 | |
| \| *VMNI: Non-invasive mechanical ventilation*  *IOT: orotracheal intubation*  *N: number of patients* \| \| --- \| | |  |

**Supplemental Table 2: Summary of clinical data of COVID-19 patients from serum cohort 5.**

|  | COVID-19 Serum samples | |
| --- | --- | --- |
| n | 48 | |
| Age (years) | 68 ± 13 | |
| Gender (F/M) | 21/27 | |
| PCR + | 48 | |
| Mild (n) | 2 | |
| Moderate (n) | 20 | |
| Severe (n) | 26 | |
| Very severe (n) | 0 | |
| Obesity (n) | 15 | |
| Cardiopathy (n) | 4 | |
| Chronic Pulmonary disease (n) | 5 | |
| Chronic Renal disease (n) | 2 | |
| Immunosuppression (n) | 3 | |
| Cancer in last 5 years (n) | 1 | |
| Pneumonia (n) | 38 | |
| Hospitalization (n) | 32 | |
| ICU income (n) | 38 | |
| Respiratory distress | 10 | |
| VMNI | 13 | |
| IOT | 3 | |
| Exitus | 2 | |
| Vaccinated (n) | 0 | |
| \| *VMNI: Non-invasive mechanical ventilation*  *IOT: orotracheal intubation*  *N: number of patients* \| \| --- \| | |  |

**Supplemental Table 3:** **Summary of clinical data of COVID-19 patients from peripheral blood mononuclear cell (PBMCs) cohort.**

|  | COVID-19 PBMCs samples |
| --- | --- |
| n | 5 |
| Age (years) | 65 ± 12 |
| Gender (F/M) | 0/5 |
| PCR + | 5 |
| Mild (n) | 0 |
| Moderate (n) | 4 |
| Severe (n) | 1 |
| Very severe (n) | 0 |
| Obesity (n) | 2 |
| Cardiopathy (n) | 2 |
| Chronic Pulmonary disease (n) | 2 |
| Chronic Renal disease (n) | 1 |
| Immunosuppression (n) | 0 |
| Cancer in last 5 years (n) | 1 |
| Pneumonia (n) | 5 |
| Hospitalization (n) | 5 |
| ICU income (n) | 0 |
| Respiratory distress | 1 |
| VMNI | 0 |
| IOT | 1 |
| Exitus | 0 |
| Vaccinated (n) | 0 |
| \| *VMNI: Non-invasive mechanical ventilation*  *IOT: orotracheal intubation*  *N: number of patients* \| \| --- \| | |

**Supplemental File 1: Proteomic identification and analysis from PBMCs from healthy donors and COVID-19 patients.**

The information obtained from the mass spectrometry analysis is summarized in this file. Each sheet contains information for one of the comparisons performed.

The parameters summarized in each of the sheets are described in the following table:

| **Column(s)** | **Parameter** | **Description** |
| --- | --- | --- |
| **A** | Accession | Uniprot accession number |
| **B** | Description | Uniprot protein description |
| **C** | Student's T-test Significant | + means signficance (p<0.05) |
| **D** | Student's T-test significant | Comparison for which a significant t-test was obtained |
| **E** | Peptides | Total number of peptides |
| **F** | Razor + unique peptides | Total razor* and unique peptides identified for a certain protein group |
| **G** | Unique peptides | Unique peptides, exclusive of that protein group |
| **H** | -Log Student's T-test p-value | -log 10 of the p value of the corresponding t-test |
| **I** | Student's T-test Difference | Difference between the average log2 protein abundances for the groups compared |
| **J** | p value | Student's t-test p value |
| **K** | Ratio | Average protein abundance ratio for the groups compared |
| **L** | Student's T-test Test statistic | Student's t-test statistic |
| **M-AV** | Protein abundance | log2 transformed protein abundance |

*Razor peptides are non-unique peptides that have been assigned to the protein group with the largest number of peptides identified.
